# Improving postural stability among people with lower-limb amputations by tactile sensory substitution

**DOI:** 10.1186/s12984-021-00952-x

**Published:** 2021-11-06

**Authors:** Lijun Chen, Yanggang Feng, Baojun Chen, Qining Wang, Kunlin Wei

**Affiliations:** 1 School of Psychological and Cognitive Sciences, Peking University, 5 Yiheyuan Road, 100871 Beijing, China; 2 Beijing Key Laboratory of Behavior and Mental Health, Beijing, China; 3 Department of Advanced Manufacturing and Robotics, College of Engineering, Peking University, Beijing, China; 4 Key Laboratory of Machine Perception, Ministry of Education, Beijing, China; 5 Peking-Tsinghua Center for Life Sciences, Beijing, China

**Keywords:** Sensory substitution, Lower-limb amputation, Postural control, Intelligent prosthesis

## Abstract

**Background:**

For people with lower-limb amputations, wearing a prosthetic limb helps restore their motor abilities for daily activities. However, the prosthesis's potential benefits are hindered by limited somatosensory feedback from the affected limb and its prosthesis. Previous studies have examined various sensory substitution systems to alleviate this problem; the prominent approach is to convert foot–ground interaction to tactile stimulations. However, positive outcomes for improving their postural stability are still rare. We hypothesized that the sensory substiution system based on surrogated tactile stimulus is capable of improving the standing stability among people with lower-limb amputations.

**Methods:**

We designed a wearable device consisting of four pressure sensors and two vibrators and tested it among people with unilateral transtibial amputations (n = 7) and non-disabled participants (n = 8). The real-time measurements of foot pressure were fused into a single representation of foot–ground interaction force, which was encoded by varying vibration intensity of the two vibrators attached to the participants’ forearm. The vibration intensity followed a logarithmic function of the force representation, in keeping with principles of tactile psychophysics. The participants were tested with a classical postural stability task in which visual disturbances perturbed their quiet standing.

**Results:**

With a brief familiarization of the system, the participants exhibited better postural stability against visual disturbances when switching on sensory substitution than without. The body sway was substantially reduced, as shown in head movements and excursions of the center of pressure. The improvement was present for both groups of participants and was particularly pronounced in more challenging conditions with larger visual disturbances.

**Conclusions:**

Substituting otherwise missing foot pressure feedback with vibrotactile signals can improve postural stability for people with lower-limb amputations. The design of the mapping between the foot–ground interaction force and the tactile signals is essential for the user to utilize the surrogated tactile signals for postural control, especially for situations that their postural control is challenged.

## Introduction

For people with amputations, wearing a prosthetic limb can help restore their motor functions and improve life quality. For fluent and adaptive motor performance, the nervous system employs a closed sensorimotor loop where efferent motor outputs are continuously coupled with afferent sensory feedback [1]. The development of typical lower-limb prosthetics, even those robotic prosthetics with actuation, focuses on the efferent control, i.e., controllability and usability of the prosthetic limb without providing the missing sensory feedback caused by amputation [2]. Studies on intelligent lower-limb prostheses have made impressive progress in adaptive control of the knee and ankle joints for walking [3–7] and even used electromyography of residual limb muscles to adjust the force or torque of prosthetic joints [8–10]. Essentially, these studies aimed to realize fluent control of the robotic prosthetics with efficiency and precision. However, supplying suitable afferent feedback for lower-limb prosthesis users is still understudied.

People with lower-limb amputations lack direct foot contact with the ground and the feedback from foot mechanoreceptors, critical for balance control [11]. With a broken sensorimotor loop, people with amputations often show poor balance and gait function with fear of falling and a high prevalence of falls [12, 13]. When a person with amputations wears a prosthesis, the residual limb physically interacts with the prosthetic sockets and provides limited haptic feedback that indirectly reflects foot–ground interaction. Augmenting this essential feedback for prosthesis wearers has the potential to close the sensorimotor control loop and subsequently improve their gait control and postural stability [14, 15].

Sensory substitution is to encode the missing sensory information and route it to the nervous system via alternative, intact sensory channels. For example, auditory and haptic feedback has been used to surrogate visual feedback for the blinded to explore the surroundings [16]. For people with upper-limb amputations, sensory substitution has been shown to provide effective sensory feedback for controlling robotic arms [17]. Previous researchers have also explored the coding of movement-related information via visual, auditory, or tactile channels for lower-limb amputations. For example, Zambarbieri, Schmid [18] used a pressure-sensing insole to estimate the center of pressure (CoP) underneath the foot and visually present the estimate to the participant. Other researchers have also used auditory feedback to deliver gait balance information and demonstrated a positive effect on gait asymmetry [19, 20]. However, both visual and auditory solutions have high demands on attention and working memory since working memory has limited processing capacities for these two sensory modalities [21]. Note that working memory is usually allocated for various cognitive tasks other than postural control. Furthermore, the auditory solutions are also practically challenging given their high demands on the surroundings' quietness. Thus, it is understandable that most researchers have turned to tactile sensory substitution for prosthetic control. The tactile feedback is typically delivered by electrotactile stimulation [22, 23] or vibrotactile stimulation [24–28], the latter being the more favorable one for people with lower-limb amputations since it is more comfortable to wear [29].

However, the potential benefits of tactile sensory substitution for lower-limb amputations have not been firmly established. Fan, Culjat [25] developed a tactile device consisting of four pneumatically controlled balloon actuators that pressed against the residue thigh of the amputated leg with a force magnitude linearly scaled by the pressure measurements from the insole of the prosthesis. They found that, based on the data from a single participant with transtibial amputation, the intensity and the order of pressing forces applied by the balloon actuators could be perceptually estimated [25, 26]. However, they did not assess the efficacy of the system in any motor task with prosthesis use. Furthermore, the large size of the balloon actuators might prevent its wide use in the population with amputations. Plauché, Villarreal [30] and Crea, Cipriani [24] used similar instrumented insoles but applied electrotactile vibrations on the thigh to inform the person with amputations about the phase transitions of gait. However, these studies only tested the device on non-disabled participants to show its feasibility and efficacy. The only study that actually examined the postural balance in people with amputations with tactile sensory substitution returned mixed or little beneficial results [27]. This study again placed four vibrators on the thigh to applied tactile stimuli contingent on the measurement of four plantar pressure sensors placed in the insole. The vibration intensity changed in proportion to the amount of plantar pressure. Three separate tasks were used to assess its effect on postural balance, including quiet standing, reaching a visual target with a cursor representation of CoP, and continuously tracking an oscillatory target with the CoP cursor. Among dozens of performance variables, only the reaction time of the CoP reaching task showed improvement with sensory substitution. In fact, the mediolateral range of CoP movements, negatively correlated with postural stability during quiet standing, increased with sensory substitution. In sum, previous researches on lower-limb amputations either did not examine the effect of tactile sensory substitution on balance performance or failed to provide a convincing beneficial effect.

These findings appear discouraging for the application of tactile sensory substitution in lower-limb amputations. However, recent studies have shown that foot–ground contact feedback delivered by directly stimulating the afferent nerves in the residuum of transtibial amputation can improve their postural stability and gait [31, 32]. Furthermore, extra tactile feedback also has been shown to improve postural control among vestibular people [33–35] and people with Parkinson’s disease [35]. We thus expect that proper design of the vibrotactile system can enhance standing balance among lower-limb amputations. Previous approaches can be improved in at least two technical aspects. First, the spatial correspondence between the foot's missing sensation and the surrogate tactile signal shall be intuitive to the prosthesis user. For instance, most studies measured plantar pressure at multiple locations underneath the foot and mapped it onto vibrotactile stimulations applied at different locations on the thigh [24, 27, 30]. It is conceivable that the motor system needs considerable training before incorporating the tactile information into the sensorimotor control loop. However, previous studies only provided limited practice before testing the effect of sensory substitution on posture and locomotion. The solution is either giving participants extensive training with the device, or making the vibrotactile stimulus simple to learn, or both. Second, previous studies typically encoded tactile stimulation as a linear function of the magnitude of plantar pressure. However, human tactile perception is a nonlinear function of stimulus amplitude, i.e., perceptual discrimination of changes deteriorates with stimulus intensity [36, 37]. Thus, a high-intensity tactile stimulus is less informative. Currently, this nonlinearity in tactile perception has not been taken into consideration to enhance the efficacy of sensory substitution. One of our previous studies also confirmed that people with amputations have more difficulty distinguishing the intensities of tactile stimuli than locating them on the skin [38].

In the present study, we designed a simplistic tactile stimulation system to provide real-time feedback of plantar pressure and tested its efficacy in improving postural stability among people with amputations and the non-disabled. We hypothesized that the improvement in encoding tactile feedback by following the principles of tactile perception psychophysics and by limiting the tactile substitution to the major direction of body sway could lead to better postural stability.

## Methods

Our sensory substitution system measured plantar pressure at four insole locations and mapped it nonlinearly to tactile intensity. Critically, to make the learning of sensory substitution easy, our system only encoded the center of pressure (CoP) excursions in the anteroposterior (AP) direction, a direction typically associated with more body sway among people with amputations than other directions [39]. Thus, we only needed to use two vibrators to encode the AP body sway. The postural stability was assessed by quiet standing under visual disturbance with the classical moving-room paradigm [40].

### Participants

We recruited seven participants with transtibial amputation as the amputation group (including six males and one female with an average age of 40.86 ± 9.40 years old) and eight non-disabled participants as the control group (including six males and two females with an average age of 23.13 ± 1.69 years). The amputation time for the participants ranged from 8 to 26 years (15.29 ± 5.99 years). Amputation was on the left side for six participants and on the right for the last participant. All participants recruited in this study had no neuromotor disease or severe cardiovascular and cerebrovascular diseases. All of them provided informed and written consent before the experiment and were paid for their participation. The Institutional Review Board of Peking University approved all procedures.

### Experiment

The whole experiment was split into two parts and completed in two successive days. The experiment would require the participant to stand quietly for 140 s in one trial, with the explicit instruction to remain stable. On day 1, all participants finished 36 trials, organized in four blocks, when wearing the sensory substitution system but without turning on the vibration. Their plantar pressure data were collected during quiet standing. These trials also served as baseline trials for estimating the range of the force loading underneath each force transducer (see below). Thus, the sensory substitution system was parameterized individually for each participant in later testing. In this way, we took the individual difference of body weight and foot conditions into consideration for designing individualized vibrotactile stimulation for each participant.

**Fig. 1:**
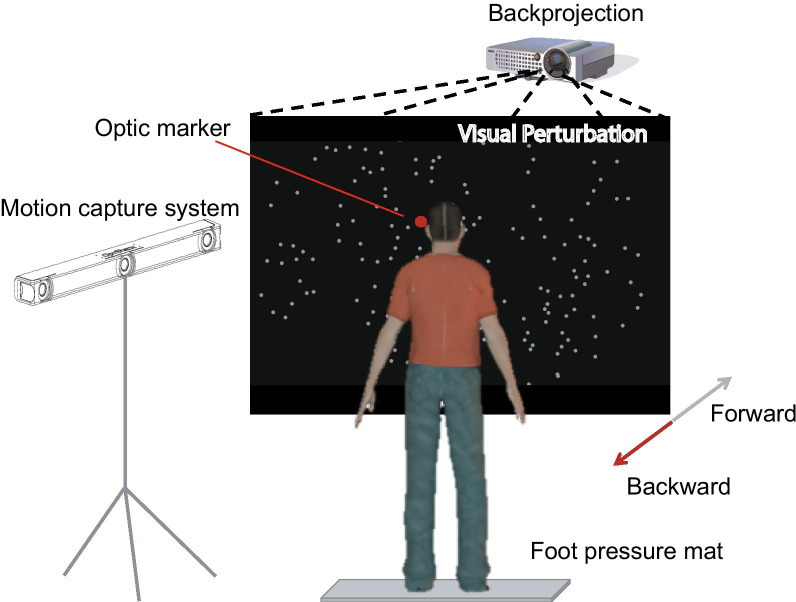
Illustration of the experimental setup for the postural stability test. The participant stands on a plantar pressure mat, facing a large projection screen. The field of view is limited to the screen by asking the participant to wear a pair of goggles. The head motion is simultaneously tracked by a motion tracking system with a marker placed at eye level. The screen displays a cloud of random dots with simulated motion in the depth direction to perturb the standing posture in the anteroposterior (AP) direction

On day 2, postural stability was evaluated with the moving-room paradigm, which perturbed the standing stance by oscillating the visual scene and examined the resulting body sway [41]. This paradigm has been used extensively to examine the dynamic stability of standing posture in different populations, including children with coordination difficulties [42] and the aging population [43]. If our participants incorporated the surrogated vibrotactile feedback into their postural control, they would be able to discount the visual disturbance more effectively when the sensory substitution system was on. The experiment was conducted in a dark room while the participant maintained a quiet standing posture 50 cm in front of a back-projection screen (Fig. 1). They were instructed to stand in a relaxed manner with two feet separated shoulder wide. The visual stimuli to provide postural disturbance were projected onto the vertically installed translucent screen by a projector (InFocus, model IN104). The viewing area was 102 cm long and 68 cm high, centered in between two eyes. Throughout the experiment, the participants wore a pair of goggles, limiting the field of view to approximately 120° wide and 60° high. Thus, the screen edge was not visible to the participant, preventing it from being served as a visual reference for stabilizing posture. The experimental setup was similar to the one used in one of our previous studies [44]. We tracked participants’ head movements throughout the experiment by an infrared motion capture system (OptiTrack, V120: Trio, Natural Point Inc.). A reflective marker was fixed on the goggle side and approximately centered in the measurement volume of the motion capture cameras. As participants stood on a plantar pressure mat (RsScan Inc., Model footscan), their foot plantar pressure and center of pressure (CoP) movements were simultaneously measured along with their head movement. The sampling frequency was set at 60 Hz for both measurements. The stimuli presentation was generated by using the Psychtoolbox package in Matlab, and data acquisition was controlled by a single customized Matlab program (Mathworks, version 2013a).

In each trial, the participants stood for 140 s, and the first 20 s were unperturbed. Then, they were perturbed by the classical moving-room paradigm in which the visual oscillatory disturbance was continuously presented to the participant [41, 45]. The stimulus consisted of 200 randomly generated dots, each with a size of 0.57 deg in diameter. The dots were randomly distributed in an annulus between 10 and 45 deg visual eccentricity [44]. No stimulus was presented in the central foveal region to avoid aliasing effects [41]. Effectively, the dots simulated a space with depth before participants’ eyes. During the experiment, the depth of the visual scene oscillated in the AP direction. This was achieved by changing the size of the dots and the distance between the dots according to visual perspective. The AP movement of the visual stimulus was sinusoidal with a certain frequency and amplitude. As the body sway was modulated by both the frequency and amplitude of the oscillation, we used three frequencies (0.1/0.3/0.5 Hz) and three amplitudes (2/4/8 cm) to cover the parameter range typically reported in the literature. This resulted in a total of nine stimulus conditions.

Both the amputation group and the control group were examined for their postural stability with and without sensory substitution. Each participant went through all the nine stimulus conditions, four trials each condition. The total 72 trials were arranged as eight trial blocks, four blocks with sensory substitution and the other four without. Each block thus consisted of 9 trials, one trial for each of the nine stimulus conditions. Trials were randomly ordered within each block. Participants were instructed to fixate at the center of the display, which was left free of moving dots with a 10° eccentricity. As the visual scene moved in the AP direction, the participant's CoP was also displaced in the same direction. To prevent fatigue, we administrated a rest of 2 to 3 min between trials and a mandatory rest of 5 min between blocks.

The whole experiment lasted about 7 h, 3.5 h each day. Participants needed to complete a total of 36 trials in 4 blocks without sensory substitution on day 1 to establish their baseline postural stability before sensory substitution. They then completed another four blocks of 36 trials on day 2 to examine the effect of sensory substitution. Note, as previous studies have not shown any habituation of visual disturbance in the moving room paradigm, we did not counterbalance the conditions between days.

### The hardware of the sensory substitution system

**Fig. 2:**
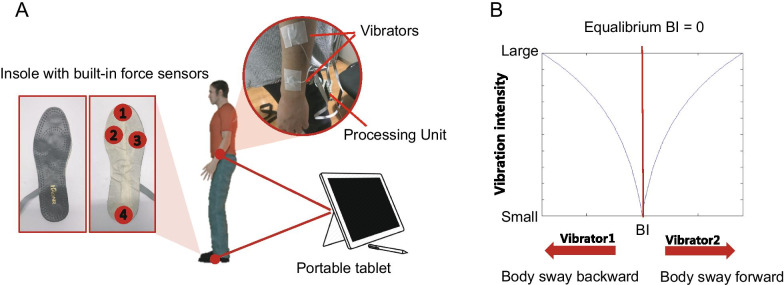
Illustration of the sensory substitution system. **A** The insole is instrumented with four thin electropiezo force sensors whose measurements are routed to a tablet for real-time data processing. The measured force determines the vibration intensity of the two tactile vibrators attached along the forearm's longitudinal axis on the same side of the insole. When participants walk or stand still, the vibration provides real-time feedback of the balance performance from the measured foot. **B** The vibration intensity follows a logarithmic relationship with the balance index (BI), which is determined by the changing force loading caused by body sway. Forward or backward body lean would lead to one vibrator working, respectively

Our sensory substitution device consisted of four electropiezo force sensors (*FlexiForce* A401, Tekscan, Inc.) and two miniaturized vibrators. We instrumented an insole with the force sensors at four critical locations, including the areas under the calcaneus tuberosity, the fourth metatarsal, the first metatarsal, and the hallux (Fig. 2A). One of our previous researches found that the force readings from these four locations could capture most of the data variance in plantar pressure during walking [8]. Since the feet size varied among participants, we customized the shape of the insole for each individual participant. The sensor was circular with a diameter of 2.54 cm and a thickness of merely 0.208 mm. The response time of the sensor was less than 5 μs with a sampling rate of 100 Hz.

The vibrotactile feedback was delivered by the two circular vibrators, which were 12 mm in diameter, 3.4 mm in height, and 1.7 g in mass. The latency of these vibrators was approximately 10 ms. They were placed along the long axis of the forearm of the amputation side (Fig. 2A). For the control participants, both the instrumented insole and the vibrators were placed on the body's left side. The two vibrators were separated by 10 cm, which was distant enough to prevent possible perceptual ambiguity across the simulated locations. The vibration amplitude and frequency were coupled together for the miniaturized vibrators. Thus, we only adjusted their vibration intensity by a pulse width modulation (PWM). The vibration intensity was modulated by the duty cycle of the PWM signal. Both the force sensors and the vibrators were connected to a tablet computer (Microsoft Surface 4) via an RS232 serial interface with a customized driver circuit. A customized Matlab application was used for real-time signal processing (Mathworks, version 2013a). The plantar pressure signals drove the vibrators in real-time with a nonlinear mapping function (see below).

### The mapping between the plantar pressure signals and the vibrotactile stimulation

**Fig. 3:**
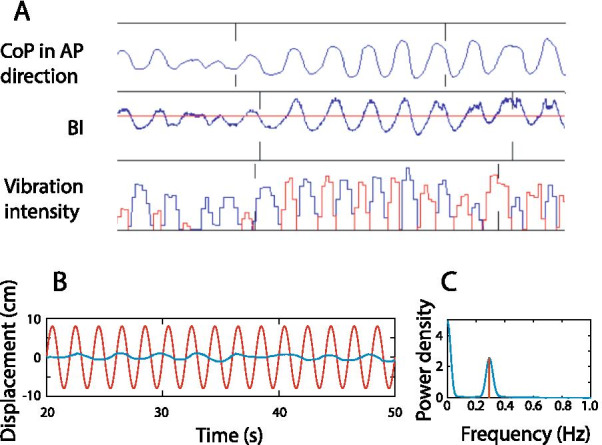
Exemplary data from the moving room paradigm. **A** An exemplary trial segment to show how the sensory substitution system works. The participant is perturbed by the oscillatory visual stimuli, resulting in large CoP displacement in the AP direction. Our system computed the *BI* index in real-time and changed the vibration intensity of the two vibrators (shown in blue and red, respectively) placed on the forearm of the participant. **B** An exemplary trial with CoP displacement (blue) and visual stimulus displacement (red) in the AP direction. The trial is shown from the 20th second to the 50th second, while in the experiment, a trial lasted 140 s. **C** The power spectral density of CoP displacement data of the same trial. The frequency of the visual stimulus here is 0.3 Hz. The power spectral density at the driving frequency was taken from the power spectral density function for each trial

The readings from the force sensors were converted into an index signifying the body weight shifts in the anterior–posterior direction. We named this index as balance index (*BI*). It was calculated as the ratio between the average force of the three force sensors in the forefoot (marked as 1 to 3 in Fig. 2A) and the force of the 4^th^ force sensor placed under the hallux:

$$BI=\frac{(F1+F2+F3)/3}{F4}$$

where F1, F2, F3, and F4 are the readings from the four force sensors, respectively. Thus, the changes in the amplitude of *BI* denote the postural sway in the AP direction (Fig. 3A, see how it coveries with CoP). If the body leans forward, the signal strength of the force sensors in the forefoot will increase while the signal strength of the 4^th^ force sensor under the heel would decrease, increasing *BI*. Conversely, a backward body sway would lead to a decrease in *BI*. The *BI* was not for quantifying postural stability but for characterizing body sway in real-time for the sensory substitution system. We estimated the average *BI* for the neutral posture when each participant was asked to stand still without any disturbance in the baseline trials on day 1. This average *BI* was defined as an equilibrium point (EP), and typically the *BI* would oscillate around each subject’s EP. The *BI* changes around the EP would be transformed into vibrotactile stimuli delivered to the forearm.

To reduce the ambiguity of vibrotactile signals, we only activated one vibrator at a time: when the *BI* was larger than the EP, the vibrator placed in the front would vibrate to signal a forward lean, and vice versa. The intensity of vibration for each vibrator was determined by the absolute difference in *BI* between the current state and the equilibrium state at EP:

$$Intensity=\frac{\mathrm{log}(BI)-{\mathrm{log}(BI}_{EP})}{\mathrm{log}{BI}_{max}}$$

where *BI*_*EP*_ is the average *BI* estimated at EP, and *BI*_*max*_ is the maximum *BI* in the forward or the backward direction estimated from the trials when the participants first encountered visual perturbation on day 1 without sensory substitution. The relation between the vibration intensity and the *BI* followed a logarithmic function (Fig. 2B). When the *BI* slightly oscillated around the equilibrium point as participants maintained a relatively neutral position, the vibrotactile feedback was weak (see an example vibration, Fig. 3B). As the *BI* deviated more from EP, the intensity would increase, approaching the maximum vibration intensity specified by the maximum *BI* estimated in the baseline trials. Thus small body sways would be more perceivable with the logarithmic transformation than a simple linear function. Correspondingly, for large body sways, the tactile stimulation is not as strong as with a linear function. We “sacrifice” the range of large signals in our tactile coding since large body sways are readily perceivable by other sensory modalities such as vision and proprioception. Furthermore, studies of human psychophysics indicated that tactile perceptual discrimination deteriorates with stimulus intensity [36, 37], suggesting that large tactile signals are less informative. Thus, our sensory substitution's encoding scheme is to highlight the feedback of small body sways but discount that of large body sways in keeping with psychophysics principles. We acknowledge that other nonlinear transformations (e.g., exponential instead of logarithmic transformation) would similarly work since they qualitatively conform to principles of perception psychophysics.

### Data analysis

We analyzed the CoP or head movements while the participant stood quietly with or without visual disturbance. All the head and CoP movement data were filtered with a zero-lag 4th-order Butterworth low-pass filter at 20-Hz cut-off frequency. For quiet standing without disturbance, we quantified the postural stability by examining the range of CoP displacement in the AP and mediolateral (ML) directions and the average CoP displacement in each trial. The range measure specifically quantified the maximum body sway, and we confirmed that it showed nearly identical changes as root mean square (RMS) of CoP, another conventional CoP measure. The RMS results were thus not reported. The average displacement was computed as the total distance of the CoP excursion divided by its duration. Thus, this variable characterized CoP stability in the AP and ML direction simultaneously.

We focused on the stability measures in the AP direction to assess postural stability under visual disturbance since the participants were visually perturbed in this direction only (see an exemplary trial, Fig. 3B). Furthermore, the majority of previous studies using the moving-room paradigm have analyzed the AP direction only. For each trial, we computed the range of CoP in the AP direction and the average displacement of CoP during the visual disturbance, similar to the analysis for quiet standing without visual disturbance. As people tend to stabilize their heads when optic flow changes with the moving-room stimuli, we also computed the head movement range. We standardized the range of head movement by dividing it with the height of each participant to minimize the effect of individual differences in body height. Given that the visual disturbance was delivered at a specific frequency in each trial, we also quantified the postural responses specific to the “driving” frequency. We first computed the power spectral density (PSD) using Welch's overlapped segment averaging estimator with eight segments overlapped by 25%. The data was windowed by a Hamming window and zero-padded. Then, the PSD at the driving frequency was obtained for each trial, showing as a response gain of visual disturbance (Fig. 3C). Thus, for all measures, i.e., movement range, average displacement, the power at the driving frequency increased in magnitude when the body sway increased. Note, all our performance measures directly reflected the amplitude of body sway, based on CoP and head movements. Though in principle, more body sway is not equivalent to less postural stability, we still regarded these measures as indicators of postural stability since we specifically required our participants to remain stationary throughout the experiment, with and without visual disturbances.

For quiet standing without visual disturbance, we conducted a two-way mixed-design ANOVA with 2 (sensory substitution on vs. off) × 2 (control vs. amputation group) for each measure. For standing with visual disturbance, we were also interested in how participants performed to different visual stimuli; thus, a 4-way mixed-design ANOVA with 3 (stimuli frequency) × 3 (stimuli amplitude) × 2 (sensory substitution on vs. off) × 2 (control vs. amputation group) was used for each measure. A Greenhouse–Geisser correction was used when the data did not meet the sphericity assumption of ANOVA. The equal variance assumption was examined by Levene’s test. Significant interactions were followed by simple main effects with Bonferroni correction.

We were also interested in the weight loading between two feet and its possible changes with the vibrotactile stimuli. Thus, we estimated the average foot pressure underneath each foot for each trial and computed the ratio between the left and the right foot for the control group and between the affected and the unaffected foot for the amputation group. For each group, we compared this ratio in trials with and without sensory substitution by using paired t-tests. Normality tests of the data were performed before the t-tests. All statistical tests were conducted by IBM SPSS Statistics for Mac, version 22 (IBM Corp., Armonk, N.Y., USA). We set the significance level at α = 0.05.

## Results

**Fig. 4:**
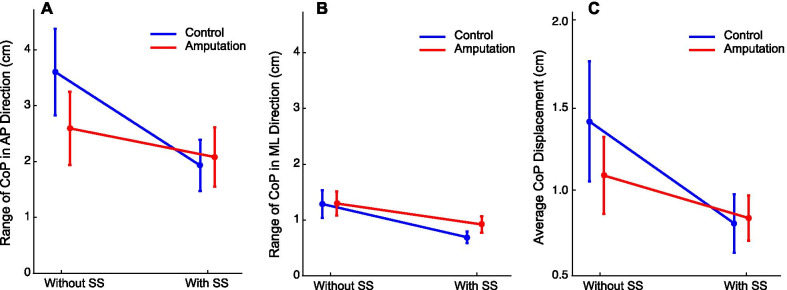
Performance variables of CoP excursion with and without sensory substitution during quiet standing. The range of CoP in the AP direction (**A**) and in the ML direction (**B**) and the average CoP displacement (**C**) within a second were plotted separately for the control and the amputation group. The error bars denote standard errors

Based on the participants’ performance on day 1, we estimated the *BI*_*max*_ that determined the vibration intensity for each individual participant. The *BI*_*max*_ was comparable between two groups (mean and standard deviation: 0.46 ± 0.12 and 0.47 ± 0.19 for the control group and the amputation group, respectively; t_13_ = − 0.176, *p* = 0.863, two-sampled t-test). We then examined the body sway without the moving-room perturbations. Three performance variables related to CoP excursions, i.e., CoP range in the AP direction, CoP range in the ML direction, and the average CoP displacement, were submitted to the two-way mixed-designed ANOVA with group as the between-subject factor and sensory substitution as the within-subject factor. We found that, for all three variables, the main effect of group was not significant (*F*_(1, 13)_ = 0.26, 0.24, and 0.21, *p* = 0.620, 0.630, and 0.66, *partial* ${\eta }^{2}$ = 0.02, 0.02, and 0.02 for the CoP range in the AP direction, in the ML direction, and average CoP displacement, respectively), but the main effect of sensory substitution was (*F*_(1, 13)_ = 17.55, 20.14, and 10.05, *p* = 0.001, 0.001, and 0.007, *partial* ${\eta }^{2}$ = 0.57, 0.61, and 0.44 for the CoP range in the AP direction, in the ML direction, and average CoP displacement, respectively). The interaction between the group and sensory substitution was significant only for the CoP range in the AP direction, indicating that the control group reduced more when the vibrotactile feedback was provided than the amputation group (*F*_(1, 13)_ = 4.91, *p* = 0.045, *partial* ${\eta }^{2}$ = 0.27). Despite that no visual disturbance was applied in the AP direction, we observed a substantially larger CoP excursion in the AP direction than in the ML direction, as reflected by the amplitudes of the CoP range (Fig. 4A vs. B). Overall, the body sway was significantly reduced with the aid of sensory substitution in two groups of participants, as reflected by reduced CoP excursion in both AP and ML directions.

**Fig. 5:**
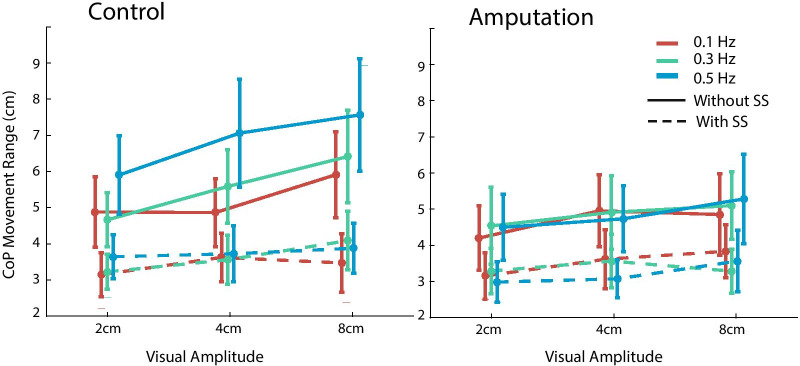
The range of CoP displacement in the AP direction plotted as a function of stimulus amplitude and frequency. The conditions with and without sensory substitution (SS) are shown in separate lines. The able-bodied control group and the amputation group are shown in the left and right panels, respectively

**Fig. 6:**
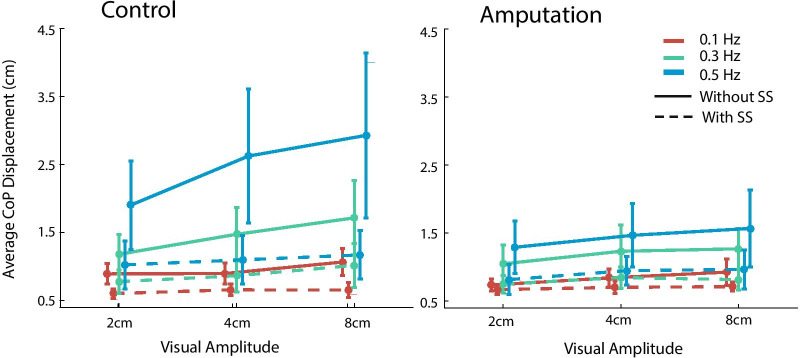
The average CoP displacement plotted as a function of stimulus amplitude and frequency. The conditions with and without sensory substitution (SS) are shown in separate lines. The control group and the amputation group are shown in the left and right panels, respectively

**Fig. 7:**
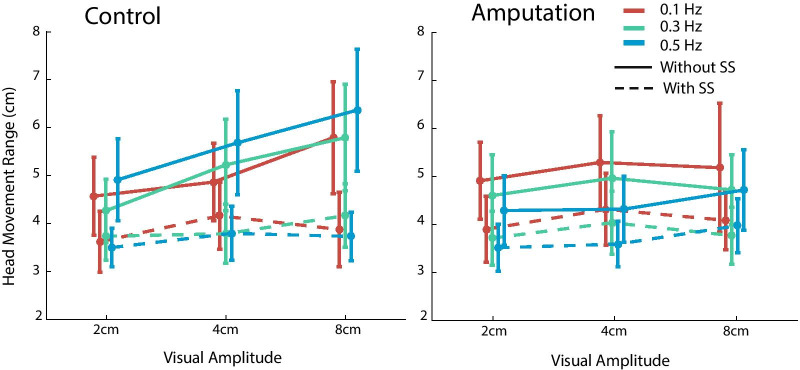
The head movement range in the AP direction plotted as a function of stimulus amplitude and frequency. The conditions with and without sensory substitution (SS) are shown in separate lines. The control group and the amputation group are shown in the left and right panels, respectively. Note the head movement range is unitless as it is normalized by dividing the participant's body height

We then examined whether the participants can perform better with sensory substitution when faced with various visual disturbances. We found that the visual disturbance modulated CoP displacement and head movement as a function of visual stimulus properties, but the body sway was reduced when the sensory substitution system was on for both groups of participants. These effects can be shown by changes in CoP displacement (Figs. 5 and 6) and head movement in the perturbed AP direction (Fig. 7).

The four-way ANOVA on the range of CoP displacement found that the main effect of group was not significant (*F*_(1, 13)_ = 0.17, *p* = 0.684, *partial* ${\eta }^{2}$ = 0.01), but the main effect of sensory substitution was highly significant (*F*_(1, 13)_ = 20.91, *p* = 0.001, *partial* ${\eta }^{2}$ = 0.62). Across groups, the CoP range before applying sensory substitution was larger than after (5.31 ± 0.75 cm v.s. 3.44 ± 0.46 cm, mean ± std. error, same below; Fig. 5). Note it appeared that the control group had a substantially larger CoP range when without sensory substitution, but this was largely due to a single participant (the fourth participant). We confirmed that removing this participant would not affect the overall results. The main effect of stimulus frequency was not significant (*F*_(2, 26)_ = 2.04, *p* = 0.151, *partial* ${\eta }^{2}$ = 0.14) but the main effect of the stimulus amplitude was (*F*_(2, 26)_ = 13.10, *p* < 0.001, *partial* ${\eta }^{2}$ = 0.50). For interaction effect, only the interaction between stimulus frequency and sensory substitution reached significance (*F*_(1.79, 23.29)_ = 6.54, *p* = 0.005, *partial* ${\eta }^{2}$ = 0.34). This interaction indicated that the benefit brought by sensory substitution was larger in the conditions with visual disturbance of a higher frequency, which was more perturbing than the conditions of lower frequencies. Simple main effects tests indicated that the effect of sensory substitution was significant for all frequency conditions (all *p*s < 0.005 after Bonferroni correction).

The CoP excursion was also characterized by its average displacement, which was submitted to the same four-way ANOVA (Fig. 6). The main effect of group was not significant (*F*_(1, 13)_ = 0.40, *p* = 0.536, *partial* ${\eta }^{2}$ = 0.03), but the main effect of sensory substitution was (*F*_(1, 13)_ = 8.94, *p* = 0.010, *partial* ${\eta }^{2}$ = 0.41). Again, the seemingly large group difference was due to large body sway of the single participant in the control group. The property of visual stimulus affected the CoP displacement with significant main effects of frequency (*F*_(2, 26)_ = 5.00, *p* = 0.015, *partial* ${\eta }^{2}$ = 0.28) and amplitude (*F*_(2, 26)_ = 6.95, *p* = 0.004, *partial* ${\eta }^{2}$ = 0.35). All the interactions failed to reach significant level except the interaction between stimulus amplitude and sensory substitution (*F*_(2, 26)_ = 4.22, *p* = 0.026, *partial* ${\eta }^{2}$ = 0.25) and the interaction between stimulus frequency and sensory substitution (*F*_(2, 26)_ = 4.87, *p* = 0.016, *partial* ${\eta }^{2}$ = 0.27). These interactions indicated that the reduction of average CoP displacement by sensory substitution was larger with increasingly large visual disturbance, either by stimulus frequency or amplitude. In fact, simple main effect indicated that the effect of sensory substitution was significant for each level of amplitude and frequency (all *p*s < 0.05 after Bonferroni correction).

Power spectrum analysis of the CoP displacement in the AP direction was used to estimate the postural response, specifically at the driving frequency of the visual stimuli. The power of CoP displacement at the driving frequency was submitted to the same four-way ANOVA. The same four-way ANOVA revealed a significant main effect of sensory substitution (*F*_(1, 13)_ = 5.08, *p* = 0.042, *partial* ${\eta }^{2}$ = 0.28), indicating that turning on the sensory substitution system reduced the COP excursion in response to the visual disturbance. The average power was 5.26 ± 2.49 and 1.52 ± 0.98 with and without sensory substitution. The main effects of group and stimulus frequency were not significant (*F*_(1, 13)_ = 0.57, *p* = 0.464, *partial* ${\eta }^{2}$ = 0.04 for group; *F*_(2, 26)_ = 0.33, *p* = 0.723, *partial* ${\eta }^{2}$ = 0.03 for stimulus frequency). The main effect of stimulus amplitude was marginally significant (*F*_(2, 26)_ = 2.89, *p* = 0.073, *partial* ${\eta }^{2}$ = 0.18). None of the interaction effects was significant except the interaction between sensory substitution and stimulus amplitude (*F*_(2,26)_ = 3.61, *p* = 0.041, *partial* ${\eta }^{2}$ = 0.22). The interaction, again, indicates that the benefit of sensory substitution was more pronounced in the conditions with larger visual amplitudes than with lower amplitudes. Simple main effects tests found that the effect of sensory substitution was significant for the 2 cm amplitude condition (*p* = 0.022) but only marginally significant for the 4 cm and 8 cm conditions (*p* = 0.050 and 0.051, respectively). Overall, the power spectrum analysis revealed that the postural response at the stimulus frequency of the visual disturbance exhibited similar changes as the overall CoP excursion.

While the CoP displacement reflects the overall body weight shifts during standing, the head movement directly reflects the body sway at eye level. We found that head movements also showed a similar benefit of sensory substitution (Fig. 7). For the head movement range, the main effect of group was not significant (*F*_(1, 13)_ = 0.05, *p* = 0.820, *partial* ${\eta }^{2}$ = 0.004). The average head movement range was comparable between the amputation group (4.20 ± 0.75 cm) and the control group (4.44 ± 0.70 cm). Importantly, the main effect of sensory substitution was significant (*F*_(1, 13)_ = 12.10, *p* = 0.004, *partial* ${\eta }^{2}$ = 0.48). Across groups, the head movement range decreased from 5.07 ± 0.65 cm to 3.88 ± 0.40 cm when the sensory substitution was used. The main effect of stimulus frequency was not significant (*F*_(2, 26)_ = 0.74, *p* = 0.487, *partial* ${\eta }^{2}$ = 0.05) but the main effect of stimulus amplitude was (*F*_(2, 26)_ = 9.66, *p* = 0.001, *partial* ${\eta }^{2}$ = 0.43). Thus, stimulus amplitude, but not stimulus frequency, modulated the head motion, similar to the results of the CoP range. All the interactions failed to reach significance except the interaction between sensory substitution and stimulus amplitude (*F*_(2, 26)_ = 3.44, *p* = 0.047, *partial* ${\eta }^{2}$ = 0.21), again indicating that the benefit of sensory substitution was more pronounced with larger visual disturbances. Simple main effect tests showed that the effect of sensory substitution was significant for each amplitude condition (all *p*s < 0.01 after Bonferroni correction). We further examined the effect of various visual stimuli by examining the reduction of head movement range by sensory substitution.

Power spectrum analysis of head movement revealed a similar pattern as the range of head movement. The power of head movement at the driving frequency of the visual stimulus was submitted to the same four-way ANOVA. The main effect of group was not significant (*F*_(1, 13)_ = 0.04, *p* = 0.841, *partial* ${\eta }^{2}$ = 0.00). The main effect of sensory substitution was marginally significant (*F*_(1, 13)_ = 3.94, *p* = 0.069, *partial* ${\eta }^{2}$ = 0.23), with an average power of 7.27 ± 3.13 and 2.95 ± 1.63 without and with sensory substitution, respectively. Both the main effects of stimulus frequency and amplitude were significant (*F*_(1.53, 19.93)_ = 5.02, *p* = 0.024, *partial* ${\eta }^{2}$ = 0.28 for frequency, *F*_(2, 26)_ = 3.63, *p* = 0.041, *partial* ${\eta }^{2}$= 0.22). The power at the driving frequency increased with stimulus amplitude, with average values of 3.08 ± 1.20, 4.79 ± 2.07, and 7.46 ± 3.48 for the 2, 4, and 8 cm conditions, respectively. Interestingly, the power at the driving frequency decreased with stimulus frequency, with average values of 8.15 ± 3.15, 5.51 ± 2.94, and 1.67 ± 0.90 for 0.1, 0.3, and 0.5 Hz conditions, respectively. None of the interactions was significant except the two-way interaction between sensory substitution and stimulus amplitude (*F*_(2, 26)_ = 3.51, *p* = 0.045, *partial* ${\eta }^{2}$= 0.21). While the larger stimulus amplitude caused larger body sway, the sensory substitution effect was larger for the larger stimulus amplitudes. Simple main effect tests showed that the effect of sensory substitution was marginally significant for each amplitude condition (*p* = 0.083, 0.066, and 0.073 for 2 cm, 4 cm, and 8 cm conditions, respectively).

A possible cause for the sensory substitution effect was that our participants could have shifted their weight to the body side without the vibrotactile stimuli to avoid the vibration sensation. In this case, the asymmetric loading on one foot might lead to reduced CoP and head movements. However, we found no evidence of changes in the symmetry of foot loadings. For the control group, the average pressure loading between the left (the vibrated side) and the right foot was 1.10 ± 0.34 and 1.06 ± 0.32 with and without vibration, respectively (paired t-tests, t_7_ = 1.29, *p* = 0.24). For the amputation group, the ratio between the affected (the vibrated side) and unaffected foot was 0.85 ± 0.22 and 0.84 ± 0.26 with and without vibration, respectively (t_6_ = 0.15, *p* = 0.89). Thus, though the participants with amputations loaded more on the non-affected side, all our participants maintained their preferred weight bearing on the two feet when the sensory substitution was on.

## Discussion

This study aimed to investigate whether people with lower-limb amputations can improve their postural stability with real-time vibrotactile feedback to surrogate their missing foot plantar pressure information. We designed a simple coding scheme for vibrotactile feedback, which only represented the body weight shifts in the AP direction with its intensity tactile psychophysics principles. We assessed the standing stability of the participants with lower-limb amputations and the non-disabled control participants during unperturbed quiet standing and during the classical moving-room paradigm. We found that both groups improved their balance control in various visual conditions when the sensory substitution was applied. The sensory substitution stabilized both the head and CoP with a large effect size (e.g., partial ${\eta }^{2}$ amounted to 0.62 and 0.48 for the range of CoP and head movements, respectively). We also found that the balance improvement brought by sensory substitution was more pronounced for more challenging conditions with larger visual disturbance. Thus, our findings suggest that closing the broken sensorimotor loop by using real-time sensory substitution can help improve postural control and, potentially, other actions that involve ground-foot interactions.

Postural control is under the simultaneous influence of multiple sensory modalities, including visual, vestibular, proprioceptive, and tactile modalities. For maintaining postural stability during standing, the nervous system adjusts the relative contributions of sensory inputs from different channels during the multisensory integration process according to the sensory precision of individual channels [46–48]. In the moving-room paradigm, the visual scene oscillates and biases the estimated standing posture, resulting in postural sway [44]. Previous researches on non-disabled participants have found that that the light touch of a fingertip on a stable surface can provide subtle tactile feedback for stabilizing posture during quiet standing and standing under visual interference [49, 50]. Vuillerme, Chenu [51] used a 6 × 6 electrotactile matrix on the tongue to provide feedback of CoP changes for the non-disabled participants and improve their postural stability when their neck proprioceptive and vestibular inputs were compromised. Our study went a step further to show that people with lower-limb amputations, similar to non-disabled participants, could also improve their postural stability against visual disturbances with vibrotactile information contingent on the plantar pressure changes. Presumably, this stabilizing effect follows the same sensory integration principles that have been repeatedly reported in different paradigms [52].

Previous studies using vibrotactile feedback to substitute foot pressure feedback have failed to show consistent benefit in postural stability (e.g., 27). We postulate that differences in the tactile coding scheme and the postural test are responsible for the discrepancy. The ease of learning and the comforts of the augmented tactile feedback presented to the human wearer were not systematically investigated until recently [53]. Our approach paid particular attention to make the tactile feedback simple. First, only bodyweight shifts, as measured by plantar pressure underneath the foot, were encoded. This is in contrast to the one-to-one signal mapping between a pressure sensor and a tactor in previous studies (e.g., 27). One-to-one mapping is technically straightforward, but it would pose a challenge for the wearer to understand tactile signals' meaning. Second, our system encodes the body sway in the anteroposterior direction, the prominent direction of instability during quiet standing. Third, we limited the two stimulators to work one at a time and used a logarithmic transfer function to use better the perceptual range of tactile stimuli [36, 37]. These signal designs help resolve the so-called neutral zone problem when people receive little tactile feedback around a neutral posture [27]. These design aspects appeared to help the participants, especially the participants with amputations who had not received direct foot contact pressure information for long, quickly learn to use surrogate sensory feedback to improve their postural control.

It is noteworthy that the benefit of our sensory substitution system manifested itself without extensive training. Our participants only familiarized themselves with the task on day 1 over 36 trials; they did not practice with sensory substitution on day 2. Previous studies on substitution of vision with tactile feedback typically required several weeks of practice time [54, 55]. We postulate that the simplicity of our vibrotactile feedback facilitated its fast adoption for postural control.

We used three stimulus frequencies (0.1/0.3/0.5 Hz) and three amplitudes (2/4/8 cm) to perturb the participant visually in our postural control task. We found that the amplitude of visual stimulus affected postural stability in every stability measure, indicating that the body sway increased with the amplitude of visual disturbance [56]. The oscillation frequency of visual disturbance showed an inconsistent effect on body sway: while time series analysis of CoP and head movements did not show an effect of stimulus frequency (for the range of data but not for the average CoP displacement), the power density at the stimulus frequency decreased for head movement but not for CoP movement. Thus, the specific gain at the oscillation frequency was damped for the head only, whose movement did not keep up with the increasing driving frequency [45].

Interestingly, no group difference of postural stability reached significance for all the performance measures investigated. We expected that people with lower-limb amputations would be perturbed more by the visual disturbances since previous studies have shown that they are more dependent on visual inputs [57–59]. However, we recognize that these studies used paradigms that reduced visual sensory feedback for the participants. Understandably, it was harder for people with amputations than the non-disabled to accommodate visual deprivation due to the loss in somatosensory feedback associated with amputation [58]. In the present study, however, we used a visual perturbation paradigm rather than visual deprivation. Furthermore, previous studies reported worse standing balance among people with amputations typically used short trials, e.g., 20 s per trial [60]. Our experiment instead used as long as 140 s per trial; thus, both groups had ample time to adapt to the visual stimuli. The other factor is that most of our participants have worn artificial limbs for more than ten years. After prolonged use of prosthesis, their CoP and head movement during quiet standing become indistinguishable from that of the non-disabled. In sum, the lack of group difference suggests that people with lower-limb amputations can effectively accommodate continuous visual disturbances.

The development of robotic artificial limbs has made continuous progress in fusing signals from various sensors for sensing the environment and the internal state of the prosthesis, but the research focus is more on intelligent control of prostheses [61]. It is equally essential to route real-time sensory feedback for the agent, i.e., the human controller, to reduce the fear of falling [12], enhance the sense of embodiment of the prosthesis [32], and better motor control [62]. The sensory augmentation for the agent can be achieved by invasive methods such as electrical peripheral nerve stimulation of the sciatic nerve [62] or noninvasive methods such as sensory substitution. As we pointed out in the introduction, substituting the missing feedback of foot–ground interaction is probably most important for people with lower-limb amputations. Still, the previous endeavors have been hampered by high demands of cognitive loads, the neglect of psychophysics of tactile perception, and inconsistent behavioral benefits. Our study has shown that these shortcomings of noninvasive sensory substitution can be overcome. It paves the way for us to integrate this method with robotic lower limbs. As most actuated lower-limb prostheses still lack afferent feedback to the user, it would be interesting to examine the outcome when our sensory substitution system integrates with these systems to achieve better human-centered close-loop control. Our study was limited to people with transtibial amputations in the laboratory environment and the test of quiet standing. Furthermore, our findings were based on CoP measurements for quantifying people’s postural stability, which can be characterized with other movement measurements. Future endeavors should be directed to testing the system among people with transfemoral amputations and via dynamic balancing tasks, such as walking on different surfaces. We expect the need for specific modifications of the signal encoding scheme for diverse movement scenarios.

## Conclusions

Using vibrotactile stimulation to substitute the missing plantar pressure information for people with transtibial amputations led to improvements in postural stability during visually-perturbed quiet standing. Both non-disabled participants and people with lower-limb amputations can benefit from sensory substitution, especially when large visual perturbations challenge their posture. Future development for sensory substitution shall consider making surrogated sensory inputs easy to comprehend and following psychophysical principles.

## Data Availability

The datasets used during the current study are available from the corresponding author on reasonable request.

## Abbreviations

CoP: Center of pressure
BI: Balance index
SS: Sensory substitution
ANOVA: Analysis of variance
